# An Evaluation Framework for Construction Quality of Bridge Monitoring System Using the DHGF Method

**DOI:** 10.3390/s23167139

**Published:** 2023-08-12

**Authors:** Jingzhou Xin, Chen Wang, Qizhi Tang, Renli Zhang, Tao Yang

**Affiliations:** 1 School of Civil Engineering, Chongqing Jiaotong University, Chongqing 400074, China; xinjz@cqjtu.edu.cn (J.X.); 18738259901@163.com (C.W.); 2 Northeast Operation Branch of Chongqing Expressway Group Co., Ltd., Chongqing 401120, China; zhangrenli@cegc.com.cn (R.Z.); yangtao@cegc.com.cn (T.Y.)

**Keywords:** bridge health monitoring, monitoring system evaluation, DHGF, comprehensive evaluation

## Abstract

Aiming at comprehensively evaluating the status of a bridge monitoring system, an evaluation framework based on the improved Delphi, analytic Hierarchy process, Grey relations analysis and Fuzzy integrated evaluation (DHGF) is selected. Firstly, the evaluation indexes for the bridge monitoring system are determined by an anonymous group discussion and expert questionnaire using the improved Delphi method. Secondly, a comparison matrix of the evaluation indexes is constructed to determine the comprehensive weight via the analytic hierarchy process. Then, based on the gray relations analysis, the albino weight function is constructed, the evaluation gray class is determined, and the single-factor fuzzy evaluation matrix is obtained. Finally, the final evaluation result was obtained by the fuzzy comprehensive evaluation. The evaluation results of a real bridge monitoring system show that the evaluation level of the monitoring system was level II, and the proposed framework could better reflect the construction and operation status of the monitoring system.

## 1. Introduction

With the long-term effect of natural factors and increasing traffic, the safety of bridge structures will inevitably deteriorate, and catastrophic accidents will occur in extreme cases. To ensure the safe operation of bridges, bridge monitoring systems have been widely established. At present, the goals of real-time monitoring, synchronous analysis, and data network sharing of monitoring systems have been gradually achieved [1,2,3,4,5]; however, the accurate diagnosis of bridge status, as the main functional goal of health-monitoring systems [6], is still difficult due to the complexity of the bridges, the randomness of external loads, and the uncertainty of the service environment. Therefore, most of the existing studies focus on the accumulation of historical data [7,8,9,10,11,12,13], the identification of external load and internal damage [14,15,16], and the assessment of overall safety [17]. For example, Jian et al. [18] automatically detected and classified a large number of fault monitoring data points by using the histogram of the relative frequency distribution of the data and the one-dimensional convolutional neural network and then comprehensively verified the selected acceleration data of the two long-span bridges. Hajializadeh et al. [19] propose a bridge damage recognition method based on deep learning, which can accurately and automatically identify the damage at different speeds and track unevenness and ambient noise. Zhang et al. [20] proposed a robustness index evaluation method based on bridge health monitoring data, which can effectively evaluate the structural state of bridges.

After the long period of operation of some monitoring facilities, there are phenomena such as a lack of basic data, damage to instruments and equipment, unstable system operation, and chaotic monitoring data. The monitoring system belongs to complex multidisciplinary cross-system engineering, and many factors, such as the coverage degree of sensor layout, the installation of monitoring equipment, and the quality of monitoring data, may cause large deviations in structural evaluation results. In this context, the evaluation of bridge monitoring systems (BHM) has become a new topic for scholars and engineers in the field. In terms of the operation state analysis of BHM systems, Li et al. [21] proposed a method for sensor fault detection using a generalized likelihood ratio and correlation coefficient and carried out manual fault sensor detection and classification verification tests on the Yangtze River Bridge, which can realize the online diagnosis of multiple sensor faults. Li et al. [22] proposed an abnormal signal detection method for the BHM system based on a two-segment deep convolutional neural network, which improved the recognition accuracy of abnormal signal patterns. Yu et al. [23] proposed a sensor state assessment and fault diagnosis method based on multi-dimensional information fusion, which can accurately obtain information from multiple sensors through the simultaneous interpretation of different sensor data. The above research focuses on the diagnosis of the monitoring status of a single sensor, and the evaluation model for the overall quality of the bridge monitoring system is rarely reported.

In view of the complexity of the operation status evaluation of BHM systems and the shortcomings of existing research, this paper proposes a DHGF-based operation status evaluation method for bridge monitoring systems and verifies the effectiveness of this method using a real-world system.

## 2. DHGF Algorithm

The DHGF comprehensive evaluation method integrates the successes of the improved Delphi method, analytic hierarchy process, gray relations analysis, and fuzzy comprehensive evaluation method [24]. The theoretical basis of the DHGF comprehensive evaluation method is the comprehensive, integrated method proposed by Qian [25] and the physics–affairs–human science (WSR) analysis method proposed by Gu, which combines disciplinary theory and human experience and knowledge and makes full use of the advantages of these two methods. To this end, the DHGF algorithm is introduced in the evaluation of bridge health monitoring systems to accurately grasp the operation status of bridge monitoring systems.

**Figure 1:**
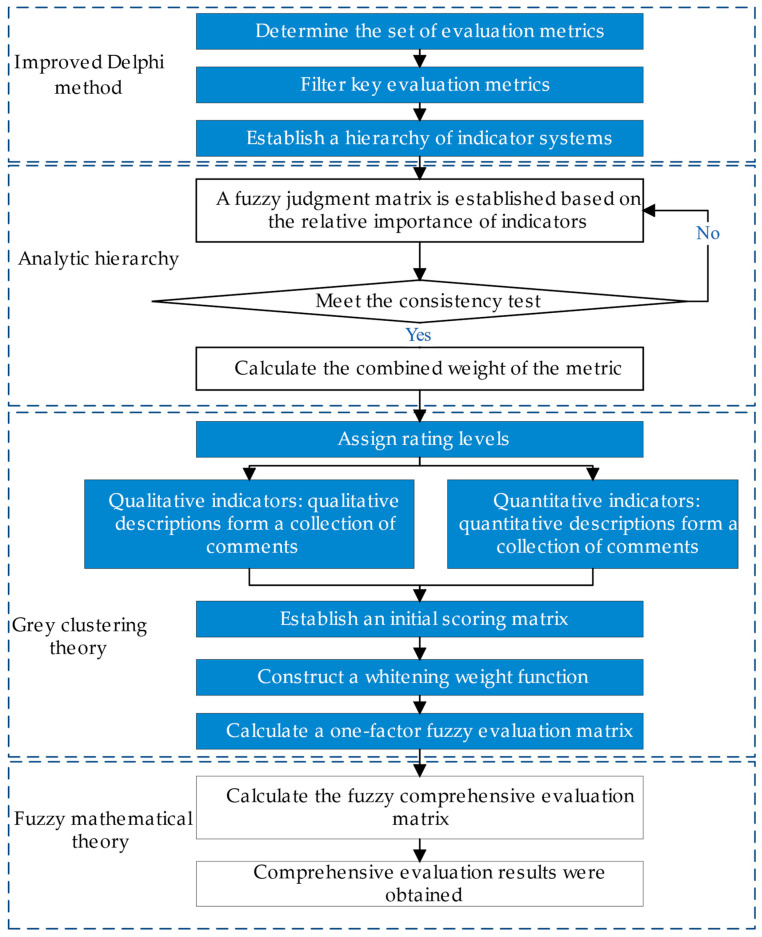
The holistic structure of the proposed method.

The basic idea of the DHGF comprehensive evaluation method is to use the improved Delphi method for anonymous discussion and statistics, optimize the evaluation indicators of the system to be evaluated, formulate the comprehensive evaluation index system, and establish a hierarchy of evaluation indicators. The analytic hierarchy process is used to calculate the combined weights of the underlying elements, and the evaluation value matrix of the evaluation index is given. The gray system theory is used to determine the evaluation gray class, and the gray evaluation coefficient is calculated to obtain the gray evaluation weight vector and weight matrix. According to the fuzzy integrated evaluation, the evaluation matrix is formed, and fuzzy calculation is carried out to obtain comprehensive evaluation results. The application of different theories and methods in different steps of the systematic evaluation can take advantage of their strengths and avoid their weaknesses, and the process of the DHGF algorithm for system state evaluation is shown in Figure 1.

## 3. Construction of Bridge Monitoring System Evaluation Model

### 3.1. Selection of Evaluation Indicators

**Figure 2:**
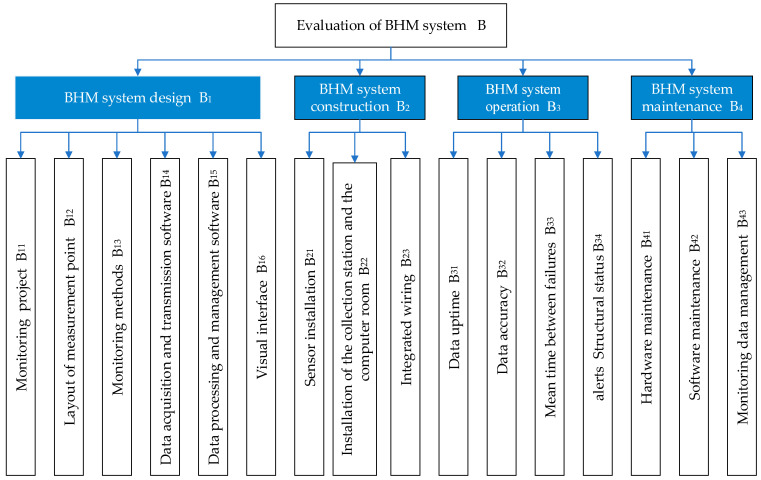
Evaluation index hierarchy of bridge monitoring system.

For the selection of evaluation indicators, the indicators are determined by means of expert group discussion and the current standard “Technical Code for Monitoring Highway Bridge Structures” (JT T 1037-2022). In order to ensure the independence of the indicators, principles based on scientificity, systematicness, accuracy, and operability are adopted. In addition, the completeness of the design function of the monitoring system, the quality control of the construction stage, the stability of the system operation status, and the timeliness of manual maintenance management are taken into account. On this basis, a hierarchical index system for the evaluation of the bridge monitoring system is constructed, and the established state evaluation index system is a three-level hierarchy, including criterion layer indicators $\left(B_{1}-B_{4}\right)$ and several index layer indicators $\left(B_{11}-B_{43}\right)$, as shown in Figure 2. The evaluation of the BHM system is mainly divided into four stages (i.e., design, construction, operation, and maintenance) to evaluate the whole lifecycle of the BHM system.

Assuming that r experts (E) participate in the evaluation and there are n evaluation indicators, $d_{hi}$ represents the score of the h expert on the index i. Then, the evaluation data of r experts on n indicators constitute an evaluation sample size matrix:

(1) $$D={\left(d_{hi}\right)}_{r\times n}=\left[\begin{array}{cccc} d_{11} & d_{12} & \ldots & d_{1n}\\ d_{21} & d_{22} & \ldots & d_{2n}\\ \ldots & \ldots & \ldots & \ldots \\ d_{r1} & d_{r2} & \ldots & d_{rn} \end{array}\right].$$

### 3.2. The Evaluation Index Weights

The importance of each indicator in reflecting the operation status of the bridge monitoring system varies. Thus, it is necessary to empower each evaluation indicator before conducting a comprehensive evaluation. In this paper, the analytic hierarchy process [24] is used to construct two pairs of judgment matrices to determine the combined weights of each index, and the determination of the judgment matrix scale is determined in Table 1. The reciprocal of the scale (1/3, 1/5, 1/7, 1/9, etc.) indicates that the importance of the two indicators is opposite to the description in the table.

A consistency test is required for the obtained judgment matrix. The results of the consistency test can exhibit the logical rationality of the judgment matrix. By this test, an awkward situation can be avoided, i.e., the indicator a is more important than the indicator b, and the indicator b is more important than the indicator c, but the indicator c is more important than the indicator a. The consistency metric is defined below:

(2) $$CI=\frac{\lambda_{\max}-m}{m-1},$$

where CR is the consistency ratio; CI is an average random consistency metric; RI is determined by the order of the matrix (see Table 2); $\lambda_{\max}$ is the maximum eigenvalue; and m represents the order of the judgment matrix.

In the process of consistency test, m is the only non-zero eigenvalues of the m-rank consensus matrix, and the maximum eigenvalues satisfy $\lambda_{\max}\geq m$; if and only if max = m, the matrix has complete consistency. Therefore, the greater the $\lambda_{\max}$ is than m, the more serious the inconsistency of the matrix. As a result, the value of $\lambda_{\max}-m$ can be used to measure the degree of inconsistency of the matrix. As for CI, the smaller the CI, the greater the consistency. It has complete consistency when CI=0, while there is good consistency when CI is close to 0.

To measure the value of CI, the random consistency index RI is introduced, which is determined by the rank of the matrix. In general, the larger the rank of the matrix, the greater the possibility of random deviation of consistency. Table 2 presents the relationship between RI and matrix rank.

Considering that the deviation of consistency may be caused by randomness, it is also necessary to compare CI and the immediate agreement index to obtain the test coefficient CR when testing whether the matrix has satisfactory consistency. The formula is as follows:

(3) $$CR=\frac{CI}{RI}.$$

When CR<0.1, it is considered that the judgment matrix has passed the consistency test, otherwise it does not have satisfactory consistency, and the judgment matrix needs to be readjusted. By calculating each judgment matrix and finding the feature vector corresponding to the largest feature value as the weight set of each indicator, the weight set can be expressed by

(4) $$W=\left\{W_{1},W_{2},\ldots ,W_{n}\right\},$$

where $W_{i}$ is the weight corresponding to indicator i. The weight vector satisfies the normalization condition, and the normalization condition is as follows:

(5) $$\sum_{i=1}^{n}W_{i}=1\&0<W_{i}<1.$$

### 3.3. Determine the Evaluation Level of the BHM System

In this paper, the evaluation level of the operation status of the BHM system is set as I, II, III, and IV, i.e., m=4, $V={\left(90,70,50,20\right)}^{T}$. Each rating corresponds to a state of the BHM system (Table 3):

● Level I indicates that the system is in an “excellent” state, where the BHM system fully meets the needs of the monitoring work and has performance beyond the requirements of “Technical Code for Monitoring Highway Bridge Structures” (JT T 1037-2022).
● Level II indicates that the system is in a “good” state, where the BHM system meets the needs of monitoring work, and the system meets the specifications of (JT T 1037-2022).
● Level III indicates that the system is in a “medium” state, where the BHM system has a few defects but basically meets the needs of monitoring work.
● Level IV means that the system is in a “poor” state, where the monitoring system defects are more obvious and cannot meet the needs of monitoring work.

According to the “Technical Code for Monitoring Highway Bridge Structures” (JT T 1037-2022), the evaluation standards for each indicator are formulated, as shown in Table 4. Among the 16 secondary evaluation indicators, two quantitative evaluation indicators are included, and the calculation of the quantitative evaluation indicators is given below.

The indicator of measurement point data integrity rate can be expressed as

(6) $$S=\left(1-\frac{\sum_{i=1}^{p}t_{i}}{P\times T}\right)\times 100\%,$$

where S is the integrity rate of the measurement point data; p is the number of fault measurement points; $t_{i}$ is the failure time of the i-th fault measurement point (take days (d) as a unit); P is the total number of measurement points; and T is the check cycle time in days (d).

The indicator MTBF is calculated by

(7) $$MTBF=\frac{\sum_{i=1}^{n}T_{i}}{\sum_{i=1}^{n}r_{i}},$$

where $T_{i}$ is the normal working time of the unit i, $r_{i}$ is the abnormal working time of unit i, and n is the total number of units in the data acquisition. Taking the integrity rate (S) of measurement point data as an example, the calculation method of the specific score of quantitative evaluation indicators is shown. From Table 4, if S is located in the interval [p,q), the corresponding score interval is [a,b), and the score (O) of the indicator is calculated by

(8) $$O=a+(b-a)\times \frac{S-p}{q-p}.$$

### 3.4. Determine the Gray Class Assessment

To comprehensively calculate the overall evaluation level of the BHM system by combining the evaluation level of each indicator, it is necessary to construct a whitening weight function to quantitatively describe the importance of the evaluation indicator. The albino weight function corresponds to an evaluation gray class for each evaluation level. Using the lowest score of the evaluation level as the threshold, the gray category is set as I, II, III, and IV according to the defined four evaluation levels; the minimum threshold of each level is 20, 50, 70, and 90, which are the gray numbers of each evaluation gray class. The corresponding whitening weight function of each level can be obtained:

For the first gray class “I” (i.e., j=1 and gray number ⊗∈[0,90,∞)), the albino weight function is written as

(9) $$f_{1}(d_{hi})=\{\begin{array}{c} \frac{d_{hi}}{90}\\ 1\\ 0 \end{array}\begin{array}{c} d_{hi}\in [0,90)\\ d_{hi}\in [90,\infty )\\ d_{hi}\in (-\infty ,0) \end{array}.$$

For the second gray class “II” (i.e., j=2, and gray number ⊗∈[0,70,140]), the albino weight function is written as

(10) $$f_{2}(d_{hi})=\{\begin{array}{c} \frac{d_{hi}}{70}\\ 2-\frac{d_{hi}}{70}\\ 0 \end{array}\begin{array}{c} d_{hi}\in [0,70)\\ \;d_{hi}\in [70,140)\\ d_{hi}\in [140,\infty ) \end{array}.$$

For the third gray class “III” (i.e., j=3, and gray number ⊗∈[0,50,100]), the albino weight function is written as

(11) $$f_{3}(d_{hi})=\{\begin{array}{c} \frac{d_{hi}}{50}\\ 2-\frac{d_{hi}}{50}\\ 0 \end{array}\begin{array}{c} d_{hi}\in [0,50)\\ d_{hi}\in [50,100)\\ d_{hi}\in [100,\infty ) \end{array}.$$

For the fourth gray class “IV” (i.e., j=4, and gray number ⊗∈[0,20,40]), the albino weight function is written as

(12) $$f_{4}(d_{hi})=\{\begin{array}{c} 1\\ \frac{40-d_{hi}}{40-20}\\ 0 \end{array}\begin{array}{c} d_{hi}\in [0,20)\\ d_{hi}\in [20,40)\\ d_{hi}\in [40,\infty ) \end{array}.$$

### 3.5. Gray Statistical Calculation

Based on the gray theory, the whitening function can find the weight $f_{j}(d_{hi})$ of each expert’s score $d_{hi}$ belonging to the j category evaluation gray class, which is denoted as $n_{ij}$. Therefore, the gray statistics for each indicator belonging to the evaluation gray category of the category j is calculated by

(13) $$n_{ij}=\sum_{h=1}^{r}f_{j}(d_{hi}).$$

The total gray statistics for each evaluation indicator are calculated by

(14) $$n_{i}=\sum_{j=1}^{k}n_{ij}.$$

The gray weights of the i-th evaluation factor and j gray category by the comprehensive r experts are calculated by

(15) $$r_{ij}=\frac{n_{ij}}{n_{i}}.$$

The univariate gray clustering coefficient matrix composed of $r_{ij}$ can be constructed by

(16) $$R=\left[\begin{array}{cccc} r_{11} & r_{12} & \ldots & r_{1m}\\ r_{21} & r_{22} & \ldots & r_{2m}\\ \ldots & \ldots & \ldots & \ldots \\ r_{n1} & r_{n2} & \ldots & r_{nm} \end{array}\right].$$

### 3.6. Fuzzy All-Round Assessment Matrix

The multiplication of the univariate weighted matrix and the gray clustering coefficient matrix is carried out to obtain the fuzzy comprehensive evaluation matrix can be constructed below [25,26]:

(17) $$B=\left[b_{1},b_{2},\ldots ,b_{m}\right]=WR=\left[w_{1},w_{2},\ldots ,w_{n}\right]\left[\begin{array}{cccc} r_{11} & r_{12} & \ldots & r_{1m}\\ r_{21} & r_{22} & \ldots & r_{2m}\\ \ldots & \ldots & \ldots & \ldots \\ r_{n1} & r_{n2} & \ldots & r_{nm} \end{array}\right],$$

where $b_{j}=\sum_{i=1}^{n}w_{i}r_{ij}$, $\sum_{j=1}^{m}b_{j}=1$.

### 3.7. Calculation of Evaluation Results

The final evaluation result Z is calculated from the fuzzy all-round evaluation matrix and the evaluation grade matrix [27]:

(18) $$Z=BV^{T}=\left(WR\right)V^{T}.$$

The final BHM system evaluation level can be obtained via Table 3.

## 4. Analysis of Example

### 4.1. Overview of the BHM System

**Figure 3:**
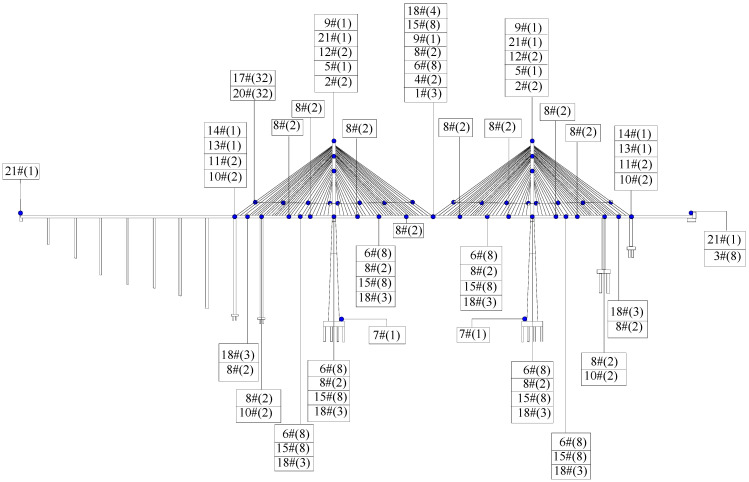
The layout of the bridge measurement point.

A cable-stayed bridge with a total length of 1001 m is employed for analysis. The BHM system is mainly composed of an automatic perception subsystem, a data acquisition and transmission subsystem, a data processing and analysis subsystem, a structural early warning and evaluation subsystem, and a visual integration subsystem, which can realize early warning in abnormal conditions. There are a total of 288 measurement points. The layout of the measurement point is shown in Figure 3. The labels in Figure 3 correspond to the type of measurement point, as shown in Table 5, and the number in parentheses is the number of measurement points.

### 4.2. Comprehensive Evaluation

The operation quality of the above BHM system was evaluated using the evaluation model established in Section 2.

#### 4.2.1. Building the Sample Matrix of Evaluation Quantities

**Figure 4:**
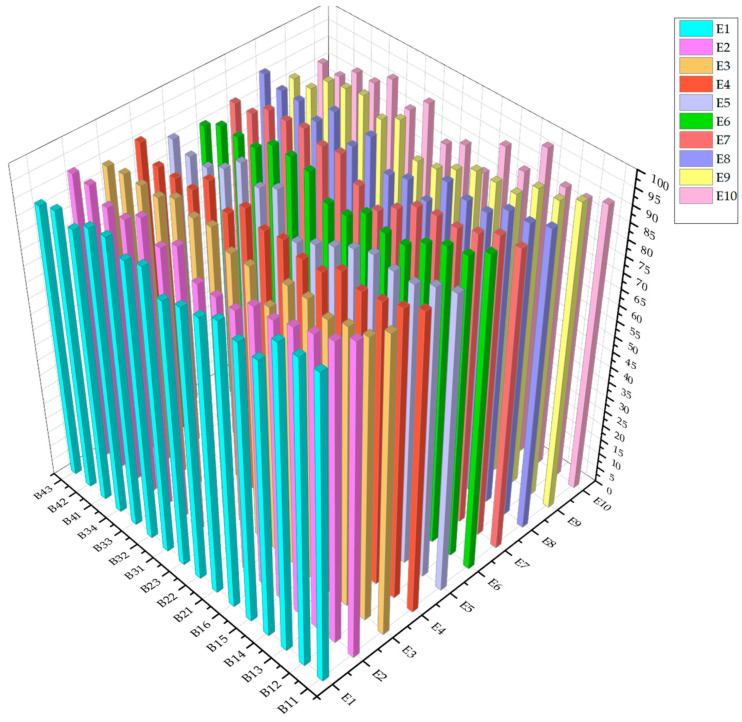
Three-dimensional view of the evaluation sample matrix.

Based on the evaluation, ten experts were invited to objectively evaluate the 16 indicators of the BHM system. The scoring range is 1–100 grades, and a sample matrix D with the size of 10 × 16 was obtained. The initial scores of the experts for each indicator are shown in Table 6 and Figure 4.

#### 4.2.2. The Weights of Evaluation Indicators

There are 16 indicators reflecting the operation status of the BHM system. To fully consider the relationship between all evaluation indicators, the weight of the evaluation indicators is calculated in hierarchical blocks [24], and the weight of each level of indicators is analyzed according to Figure 1.

The four indicators of the criterion layer are taken as examples to present the process of weights allocation: First, in comparing design ($B_{1}$) and construction ($B_{2}$), $B_{1}$ is more important than $B_{2}$, and the degree of importance is slightly more important, which is recorded as scale 2; in comparing $B_{1}$ and running ($B_{3}$), $B_{1}$ is more important than *B*_3_ and the degree of importance is slightly more important, which is denoted as scale 2; in comparing $B_{1}$ and $B_{4}$, $B_{1}$ is more important than maintenance ($B_{4}$), and the degree of importance is clearly important, which is recorded as scale 4. Then, in comparing *B*_2_ and $B_{3}$, they are of similar importance and are recorded as scale 1; in comparing with $B_{2}$ and $B_{4}$, $B_{2}$ is more important than $B_{4}$, and the degree of importance is slightly more important, which is recorded as scale 2. Finally, in comparing $B_{3}$ and $B_{4}$, $B_{3}$ is more important, and the degree of importance is slightly more important, which is denoted as scale 3. The judgment matrix between the indicators of the criterion layer is shown in Table 7. Based on Equations (2) and (3), the consistency ratio is calculated. According to Table 2, due to CI=(4.02−4)/(4−1)=0.0067, CR=0.0067/0.89=0.0075<0.1, it can be seen that there is no logical error in the judgment matrix, and the weights of the $B_{1}$, $B_{2}$, $B_{3}$, and $B_{4}$ layers are shown in the eigenvalue corresponding to the indicators in Table 7. Similarly, the final combination weights of the index layer are displayed in Table 8.

Then, the consistency for weights of the criterion layer was checked:

$$\sum_{i=1}^{4}W_{i}=0.4515+0.2257+0.2507+0.1007=1.0,$$

for the weights of the index layer:

$$\sum_{i=1}^{16}W_{i}=0.0939+0.0939+0.0325+\ldots +0.0258+0.0517=1.0.$$

Obviously, the combined weights of the above standard layer and index layer meet the normalization test.

**Figure 5:**
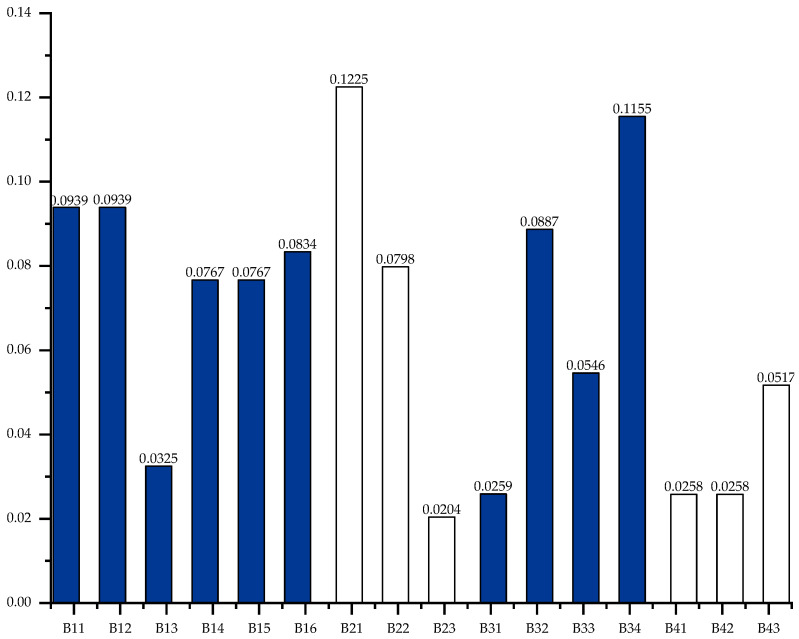
Index layer weight diagram.

The weights of the index layer are shown in Figure 5. It can be seen that the five most important indicators accounting for the heaviest proportion are $B_{21}$, $B_{34}$, $B_{11}$, $B_{12}$, $B_{32}$, and $B_{16}$.

#### 4.2.3. Gray Statistical Calculation

Taking the weight of the BHM evaluation indicator $B_{11}$ for the first gray group as an example, the gray group statistics are calculated from Equation (13):

$$n_{11,1}=f_{1}\left(98\right)+f_{1}\left(100\right)+f_{1}\left(96\right)+\ldots +f_{1}\left(98\right)+f_{1}\left(92\right)=10.0000.$$

Similarly, the evaluation indicator $B_{11}$ of gray statistics for gray groups 2, 3, and 4 is calculated $n_{11,2}=6.1857$, $n_{11,3}=0.6600$, and $n_{11,4}=0$.

The gray statistics matrix calculated for the 16 secondary evaluation indicators belonging to each gray category is as follows:

$$n_{ij}=\left[\begin{array}{rrrr} 10.0000 & 6.1857 & 0.6600 & 0.0000\\ 10.0000 & 6.5429 & 1.1600 & 0.0000\\ 10.0000 & 6.7143 & 1.4000 & 0.0000\\ 9.9667 & 7.0000 & 1.8000 & 0.0000\\ 9.9556 & 7.1429 & 2.0000 & 0.0000\\ 9.9445 & 7.1286 & 1.9800 & 0.0000\\ 9.5001 & 7.7857 & 2.9000 & 0.0000\\ 9.5334 & 7.7429 & 2.8400 & 0.0000\\ 9.4445 & 7.8571 & 3.0000 & 0.0000\\ 10.0000 & 6.8571 & 1.6000 & 0.0000\\ 9.8446 & 7.2714 & 2.1800 & 0.0000\\ 10.0000 & 6.7143 & 1.4000 & 0.0000\\ 9.8779 & 7.2143 & 2.1000 & 0.0000\\ 9.8556 & 7.2857 & 2.2000 & 0.0000\\ 9.7778 & 7.4000 & 2.3600 & 0.0000\\ 9.7780 & 7.3517 & 2.3000 & 0.0000 \end{array}\right]$$

The gray statistics of the indicator $B_{11}$ is calculated by Equation (14):

$$n_{11}=n_{11,1}+n_{11,2}+n_{11,3}+n_{11,4}=16.8457.$$

The evaluation indicator $B_{11}$ for each gray class weight can be obtained:

$$\begin{array}{l} r_{11,1}=\frac{n_{11,1}}{n_{11}}=0.5936,\;r_{11,2}=\frac{n_{11,2}}{n_{11}}=0.3672,\;\\ r_{11,3=}\frac{n_{11,3}}{n_{11}}=0.0392,{\;r}_{11,4}=0 \end{array}$$

By analogy, the univariate gray clustering matrix R for all indicators of the index layer is as follows:

$$R=\left[\begin{array}{cccc} 0.5936 & 0.3672 & 0.0392 & 0\\ 0.5649 & 0.3696 & 0.0655 & 0\\ 0.5521 & 0.3707 & 0.0773 & 0\\ 0.5311 & 0.3730 & 0.0959 & 0\\ 0.5213 & 0.3740 & 0.1047 & 0\\ 0.5219 & 0.3741 & 0.1039 & 0\\ 0.4709 & 0.3857 & 0.1437 & 0\\ 0.4739 & 0.3849 & 0.1412 & 0\\ 0.4652 & 0.3870 & 0.1478 & 0\\ 0.5418 & 0.3715 & 0.0867 & 0\\ 0.5102 & 0.3768 & 0.1130 & 0\\ 0.5521 & 0.3707 & 0.0773 & 0\\ 0.5147 & 0.3759 & 0.1094 & 0\\ 0.5096 & 0.3767 & 0.1137 & 0\\ 0.5005 & 0.3788 & 0.1208 & 0\\ 0.5031 & 0.3785 & 0.1183 & 0 \end{array}\right]$$

#### 4.2.4. Fuzzy Comprehensive Assessment Matrix

The fuzzy comprehensive evaluation matrix of the BHM system is calculated by combining the weight matrix and the univariate fuzzy evaluation matrix using Equation (17):

B=W×R=(0.5161, 0.3719, 0.1013, 0)

The comprehensive clustering coefficients of the four gray groups of the BHM system are 0.5161, 0.3719, 0.1013, and 0.

#### 4.2.5. Calculate the Results of the Overall Evaluation

The comprehensive evaluation results are calculated by the fuzzy all-around evaluation matrix and the evaluation grade matrix by Equation (18), and the final comprehensive evaluation score is Z.

The comprehensive evaluation results of the BHM system are

$$Z=BV^{T}=83.9217\in \left(70,90\right)$$

From Table 3, the BHM system belongs to evaluation level “II”.

## 5. Conclusions

To accurately grasp the operation status of the BHM system, this paper establishes an evaluation framework based on the DHGF comprehensive evaluation method. The feasibility of the proposed framework is verified by a real-world BHM system, and some main conclusions are summarized below:

The DHGF comprehensive evaluation method integrates the advantages of the improved Delphi method, the analytic hierarchy process, the gray theory, and the fuzzy comprehensive evaluation method, which can effectively meet the needs of qualitative and quantitative evaluation. Specifically, the improved Delphi method can comprehensively consider the opinions of multiple experts and avoid one-sidedness caused by personal subjective factors. Meanwhile, the analytic hierarchy process can comprehensively consider the logical relationship between the importance of multiple evaluation indicators when determining the weight of evaluation indicators. In addition, the gray theory can effectively divide the evaluation levels of each evaluation indicator according to the principle of the maximum gray clustering coefficient and effectively display the influence of each indicator on different evaluation levels; and the fuzzy comprehensive evaluation method can transform qualitative and quantitative analysis into the final quantitative evaluation results;

The evaluation of the operation status of the BHM system is a comprehensive evaluation problem involving multiple indicators. The improved Delphi method is employed to determine the evaluation indicators of the BHM system, and the evaluation indicator system is established, which can reflect the interrelationship between the indicators well. On this basis, the 16 proposed evaluation indicators can depict the operation status of the BHM system well, which is scientific, comprehensive, and feasible;

The case study shows that the proposed indicators are detailed and reasonable, and the comprehensive evaluation framework is effective and feasible. By this framework, the complex indicators of the BHM system can be integrated, and the qualitative and quantitative evaluation can be realized. Therefore, a comprehensive evaluation of the operation status of the BHM system is achieved, and the evaluation results accurately reflect the operation status of the BHM system.

This paper provides a framework for the evaluation of the BHM system and presents the evaluation process through a new BHM system. In this framework, the evaluation indicators (e.g., data quality) can be flexibly adjusted to evaluate the in-service BHM system to achieve more accurate evaluation results. 

## Figures and Tables

**Table 1:** Determining the scale and meaning of the matrix.

Scale	Meaning
1	Indicator a is just as important as indicator b
3	Indicator a is slightly more important than indicator b
5	Indicator a is more important than indicator b
7	Indicator a is important compared to indicator b
9	Indicator a is particularly important compared to indicator b
The median value	The median of the above scales (2, 4, 6, 8)

**Table 2:** Reference table of average consistency indicators.

Matrix Rank	1	2	3	4	5	6	7	8	9	10
RI	0	0	0.58	0.90	1.12	1.24	1.32	1.41	1.45	1.49

**Table 3:** Evaluation grades and score divisions of BHM systems.

Evaluation Level	I	II	III	IV
Hierarchical	[90, 100]	[70, 90)	[50, 70)	[20, 50)

**Table 4:** Indicator evaluation sets.

**Index**	I [90–100]	II [70–90)	III [50–70)	IV [20–50)
Monitoring project	The monitoring project exceeds the specifications	Monitor whether the project meets the specifications	Most of the monitoring projects meet the specifications	The monitoring project basically does not meet the requirements of the specification
Layout of measurement point	The layout of the measurement point accurately meets the needs of monitoring work, and there are redundant measurement points in key parts	The layout of the measurement point meets the needs of monitoring work, and there are redundant measurement points in key parts	The layout of the measurement points basically meets the needs of monitoring work	The layout of the measurement points basically does not meet the needs of the monitoring work
Monitoring methods	All sensor parameters meet the requirements, the monitoring method is suitable, and the sampling frequency meets the requirements	More than 90% of the sensor parameters meet the requirements, the monitoring method is appropriate, and the sampling frequency meets the requirements	More than 80% of the sensor parameters meet the requirements, the monitoring method is basically suitable, and the sampling frequency basically meets the requirements	The sensor parameters meet the requirements, the monitoring methods are mostly inappropriate, and the sampling frequency mostly does not meet the requirements
Acquisition and transmission software	The indicators are fully functional and exceed the needs of monitoring work	The indicators are fully functional to meet the needs of monitoring	The indicators have basic functions and basically meet the needs of monitoring work	The indicators are not fully functional and do not meet the needs of monitoring
Data processing and management software	The indicators are fully functional and exceed the needs of monitoring work	The indicators are fully functional to meet the needs of monitoring	The indicators have basic functions and basically meet the needs of monitoring work	The indicators are not fully functional and do not meet the needs of monitoring
Visual interface	The layout of the visual interface is very clear and reasonable, which can intuitively reflect data changes, and the response time of the operation is timely, and mobile software is available	The layout of the visual interface is clear and reasonable, which can intuitively reflect data changes, and the response time of the operation is timely, and mobile software is available	The layout of the visual interface is reasonable, which can reflect data changes, and the response time of the operation is acceptable, and mobile software is not available	The layout of the visual interface is chaotic, which does not clearly reflect data changes, operation response times are too long, and mobile software is not available
Sensor installation	Each measurement point is stable and has excellent working environment and very good protection measures	Each measurement point is stable, has a good working environment, and good protection measures	Each measurement point is stable and has an acceptable working environment and general protection measures	Each measurement point is not in a stable position and has a poor working environment and no protective measures
Installation of the collection station and the computer room	The indicator has a reasonable position and very stable installation, which is very neat and beautiful, meets the process requirements, and the terminal contact is good	The indicator has a reasonable position and stable installation, which is neat and beautiful, in line with process requirements, and the terminal contact is good	The indicator has a reasonable position, and the installation is relatively stable, which is relatively neat and basically meets the process requirements, and the terminal contact is good	The indicator does not have a reasonable position, the installation is not stable, which does not meet the process requirements, and the terminal contact is poor
Integrated wiring	The wiring is specified and is clearly marked, beyond the requirements of specifications	The wiring is relatively specified, has a relatively clear mark, and satisfies the requirements of specifications	The wiring meets the requirements of use, which has marks but falls below the specifications	The wiring is cluttered and has no clear marks
Data uptime	S ≥ 95%	90% ≤ S ≤ 95%	85% ≤ S ≤ 90%	S ≤ 85%
Data accuracy	The BHM system data are in good agreement with the manual observation data	The BHM system data are consistent with the manual observation data	The BHM system data are basically consistent with the manual observation data	The BHM system data do not match the manual observation data
Mean time between failures	MTBF ≥ 99%	99% ≤ MTBF ≤ 95%	90% ≤ MTBF ≤ 95%	MTBF ≤ 90%
Structural status alerts	The upload, analysis, and submission of the collected data are very timely; structural safety alarms are very accurate, and almost no false positives	The upload, analysis, and submission of the collected data are timely, accurate alarms for structural safety issues, and the probability of false positives is within an acceptable range	The upload, analysis, and submission of the collected data are timely, the structural safety alarm is more accurate, and there are false positives, which do not affect the use of the system	The upload, analysis, and submission of the collected data are not timely, the alarm of structural safety is inaccurate, or the system’s alarm does not work if there is a major security problem in the structure, and the probability of false positives is high
Hardware maintenance	The maintenance is very well and on time	The maintenance is well and on time	The maintenance is relatively on time	The maintenance is not on time
Software maintenance	The maintenance is very well and on time	The maintenance is well and on time	The maintenance is relatively on time	The maintenance is not on time
Monitoring data management	The monitoring data are regularly reported in detail, exceeding the requirements of the current norms	The monitoring data are regularly reported in detail, meeting the requirements of current norms	There is a slight delay in the formation of the monitoring report, and the content basically meets the requirements of the current norms	Reports are not produced regularly

**Table 5:** Labels correspond to the types of measurement points.

Label of Measurement Point	Type of Measurement Point	Number of Measurement Points
1#	Ambient temperature and humidity measurement point	3
2#	Temperature and humidity measurement point in the Sota anchoring area	4
3#	Vehicle load measurement point	8
4#	Bridge deck wind speed and wind direction measurement point	2
5#	Wind speed and direction measurement point at the top of the tower	2
6#	Structural temperature measurement point	56
7#	Ground motion measurement point	2
8#	Main beam deflection measurement point	34
9#	Lateral displacement measurement point of main beam	1
10#	Seat shift measurement point	8
11#	Beam end displacement measurement point	4
12#	Offset measurement point at the top of the tower	4
13#	Horizontal angle measurement point at beam end	2
14#	Vertical angle measurement point of beam end	2
15#	Main beam strain measurement point	40
16#	Tower column strain measurement point	16
17#	Cable-stayed cable force measurement point	32
18#	Main beam vibration measurement point	28
19#	Vibration measurement point at the top of the tower	4
20#	Cable vibration measurement point	32
21#	Video surveillance measurement points	4

**Table 6:** Initial expert scoring matrix.

$d_{li}$	$B_{11}$	$B_{12}$	$B_{13}$	$B_{14}$	$B_{15}$	$B_{16}$	$B_{21}$	$B_{22}$	$B_{23}$	$B_{31}$	$B_{32}$	$B_{33}$	$B_{34}$	$B_{41}$	$B_{42}$	$B_{43}$
$E_{1}$	98	98	98	89	90	92	89	88	86	92	90	93	92	88	90	88
$E_{2}$	100	96	94	92	90	90	85	85	85	92	88	93	89	89	92	92
$E_{3}$	96	91	90	88	90	90	80	88	88	92	91	93	90	90	90	89
$E_{4}$	96	93	91	90	92	88	88	90	89	92	87	93	87	87	87	91
$E_{5}$	95	93	90	90	91	89	86	83	80	92	89	93	88	85	85	87
$E_{6}$	100	96	95	92	88	88	90	86	86	92	93	93	89	89	89	86
$E_{7}$	96	96	93	91	91	90	86	82	86	92	91	93	92	92	88	88
$E_{8}$	96	94	94	90	90	92	83	86	84	92	86	93	87	90	90	92
$E_{9}$	98	95	95	90	90	90	87	84	83	92	89	93	92	91	86	86
$E_{10}$	92	90	90	98	88	92	81	86	83	92	87	93	89	89	85	86

**Table 7:** Criterion layer judgment matrix of BHM system.

**Criterion Layer**	BHM System Design $B_{1}$	BHM System Construction $B_{2}$	BHM System Operation $B_{3}$	BHM System Maintenance $B_{4}$	Eigenvalue	$\lambda_{max}$
BHM system design $B_{1}$	1	2	2	4	0.4515	4.02
BHM system construction $B_{2}$	1/2	1	1	2	0.2257	
BHM system operation $B_{3}$	1/2	1	1	3	0.2507	
BHM system maintenance $B_{4}$	1/4	1/2	1/3	1	0.1033	

**Table 8:** Evaluation index system and weight of BHM system.

Target Layer	Criterion Layer	Weights of Criterion Layer	Index Layer	Weights of Index Layer	Index Layer Combination Weights
BHM system evaluation	BHM system design $B_{1}$	0.4515	Monitoring project $B_{11}$	0.2080	0.0939
			Layout of measurement point $B_{12}$	0.2080	0.0939
			Monitoring methods $B_{13}$	0.0719	0.0325
			Data acquisition and transmission software $B_{14}$	0.1699	0.0767
			Data processing and management software $B_{15}$	0.1699	0.0767
			$\mathrm{Visual}\;\mathrm{interface}\;B_{16}$	0.1848	0.0834
	BHM system construction $B_{2}$	0.2257	$\mathrm{Sensor}\;\mathrm{installation}\;B_{21}$	0.5559	0.1255
			Installation of the collection station and the computer room $B_{22}$	0.3537	0.0798
			Integrated wiring $B_{23}$	0.0904	0.0204
	BHM system operation $\;B_{3}$	0.2507	$\mathrm{Data}\;\mathrm{uptime}\;B_{31}$	0.1033	0.0259
			$\mathrm{Data}\;\mathrm{accuracy}\;B_{32}$	0.2179	0.0887
			Mean time between failures $B_{33}$	0.2179	0.0546
			Structural status alerts $B_{34}$	0.4609	0.1155
	BHM system maintenance $B_{4}$	0.1007	Hardware maintenance $B_{41}$	0.2500	0.0258
			Software maintenance $B_{42}$	0.2500	0.0258
			Monitoring data management $B_{43}$	0.5000	0.0517

## Data Availability

The data presented in this study are available on request from the corresponding author.
